# Lineage tracing in human tissues

**DOI:** 10.1002/path.5911

**Published:** 2022-05-05

**Authors:** Calum Gabbutt, Nicholas A Wright, Ann‐Marie Baker, Darryl Shibata, Trevor A Graham

**Affiliations:** ^1^ Centre for Genomics and Computational Biology, Barts Cancer Institute, Barts and the London School of Medicine and Dentistry Queen Mary University of London London UK; ^2^ Centre for Evolution and Cancer Institute of Cancer Research Sutton UK; ^3^ London Interdisciplinary Doctoral Training Programme (LIDo) London UK; ^4^ Keck School of Medicine University of Southern California Los Angeles CA USA

**Keywords:** lineage tracing, clonal dynamics, clonality analysis, adult stem cells, intestinal stem cells, haemopoietic stem cells, colon, DNA sequencing, *in situ* hybridisation, quantitative methods

## Abstract

The dynamical process of cell division that underpins homeostasis in the human body cannot be directly observed *in vivo*, but instead is measurable from the pattern of somatic genetic or epigenetic mutations that accrue in tissues over an individual's lifetime. Because somatic mutations are heritable, they serve as natural lineage tracing markers that delineate clonal expansions. Mathematical analysis of the distribution of somatic clone sizes gives a quantitative readout of the rates of cell birth, death, and replacement. In this review we explore the broad range of somatic mutation types that have been used for lineage tracing in human tissues, introduce the mathematical concepts used to infer dynamical information from these clone size data, and discuss the insights of this lineage tracing approach for our understanding of homeostasis and cancer development. We use the human colon as a particularly instructive exemplar tissue. There is a rich history of human somatic cell dynamics surreptitiously written into the cell genomes that is being uncovered by advances in sequencing and careful mathematical analysis lineage of tracing data. © 2022 The Authors. *The Journal of Pathology* published by John Wiley & Sons Ltd on behalf of The Pathological Society of Great Britain and Ireland.

## Introduction

All the somatic cells in a human trace their ancestry back to the zygote. It is becoming increasingly clear that healthy normal tissue carries a high burden of somatic DNA mutations [1, 2, 3, 4] and epigenetic mutations [5] (hereafter epimutations) that accrue over the course of a person's lifetime, causing the genomes of extant lineages of cells to diverge. The hierarchical nature of tissue organisation, whereby differentiated cells are derived from a small number of multipotent stem cells, cause tissues to be a patchwork of different spatially‐segregated clones, each derived from a distinct ancestor stem cell—in this sense, our bodies are mosaics of somatic mutants. The pattern of somatic mutations in a tissue—that is the distribution of sizes of clones delineated by these somatic mutations and the spatial location of clones—is therefore a direct consequence of the dynamical process of cell birth, death, and lineage replacement in that tissue. Analysis of the pattern of somatic mutations provides a serendipitous window into human cell dynamics *in vivo*.

Further, recent studies (reviewed in [6]) have found that gene mutations commonly found in cancers that are thought to be functional for cancer development, termed *driver* mutations, are also present in a surprisingly high number of morphologically normal cells. Exploring how clonal populations evolve in ostensibly normal human tissue is an important step to understanding the earliest steps in cancer evolution, namely how one of these ‘cancer‐primed’ cells outcompetes its neighbours, clonally expands to colonise surrounding tissue, and eventually transforms into malignancy.

Lineage tracing, a general term for methods to detect parent–daughter relationships between cells using clonal markers, presents a powerful tool for probing the clonal dynamics of tissue. In mouse models, the use of experimentally induced fate markers [7] have allowed the complex and variable dynamics of various tissues to be elucidated (e.g. skin [8], colon [9, 10], and breast [11]). However, such methods are inappropriate for use in humans and therefore researchers are forced to rely on somatic lineage tracing markers. The basic principle is that the (epi)genomes of recently‐related cells are expected to be more similar than those of more distantly related cells, and so, more generally, measurements of the (epi)genetic differences between cells allow for the reconstruction of their clonal relationships. To illustrate this principle, consider two cells taken from distant locations within an individual's body—say, brain and bowel—whose most recent common ancestor arose early during embryogenesis. Every time a cell divides it accrues ∼1–10 point mutations [12, 13, 14] across the ∼3 billion base pairs within its genome. Given this very low probability that any specific base will be mutated, the probability that two randomly selected cells have independently developed the same mutation is extremely low. Consequently, the vast majority of somatic mutations will not be shared between these distantly related cells. In contrast, as discussed later, consider the genomes of two cells selected from the same colonic crypt. Because of the rapid turnover of cells in the colon fuelled by a small number of stem cells in intense competition to retain a place in the niche, the two cells will share the vast majority of their somatic mutations, reflecting the short time since their recent common ancestor.

Importantly, lineage tracing provides a powerful lens to study the dynamics of adult stem cell divisions, despite stem cells typically being rare in tissues. This is because of the rapid turnover of many tissue types [15]: this indicates that approximately every week in the intestine, a few divisions separate (short‐lived) differentiated cells from their stem cell parent [16, 17], and so the majority of somatic mutations in a differentiated cell are those that were acquired by its stem cell ancestor. This effect is even more pronounced when considering that measurements of (epi)genetic alterations have relatively low sensitivity. Sequencing methods are biased to detect near‐clonal (high frequency) alterations, which were present in the stem cell and ‘amplified’ in the population via the production of multiple differentiated cell progeny, whereas newly‐acquired mutations in a single or a small number of differentiated cells will be at low frequency and are unlikely to be detected. In this way, the measurement of all the cells in a single clonal unit (e.g. a colon crypt) is informative of the behaviour of the stem cell population that underpins that unit.

In the following sections, we review the long history of lineage tracing methodologies applied to human tissues and offer an introduction to the mathematical principles used to interpret these data. As sensitivity to detect somatic mutations has improved, so has the resolution (both over time and space) of our derived understanding of somatic cell dynamics.

## Early lineage tracing studies relied on germline mutations or clonal mosaicism

Early lineage tracing techniques in the context of human biology relied on the observation of whether a single clonal marker was shared by a given population of cells but was absent in distant cells.

**Figure 1:**
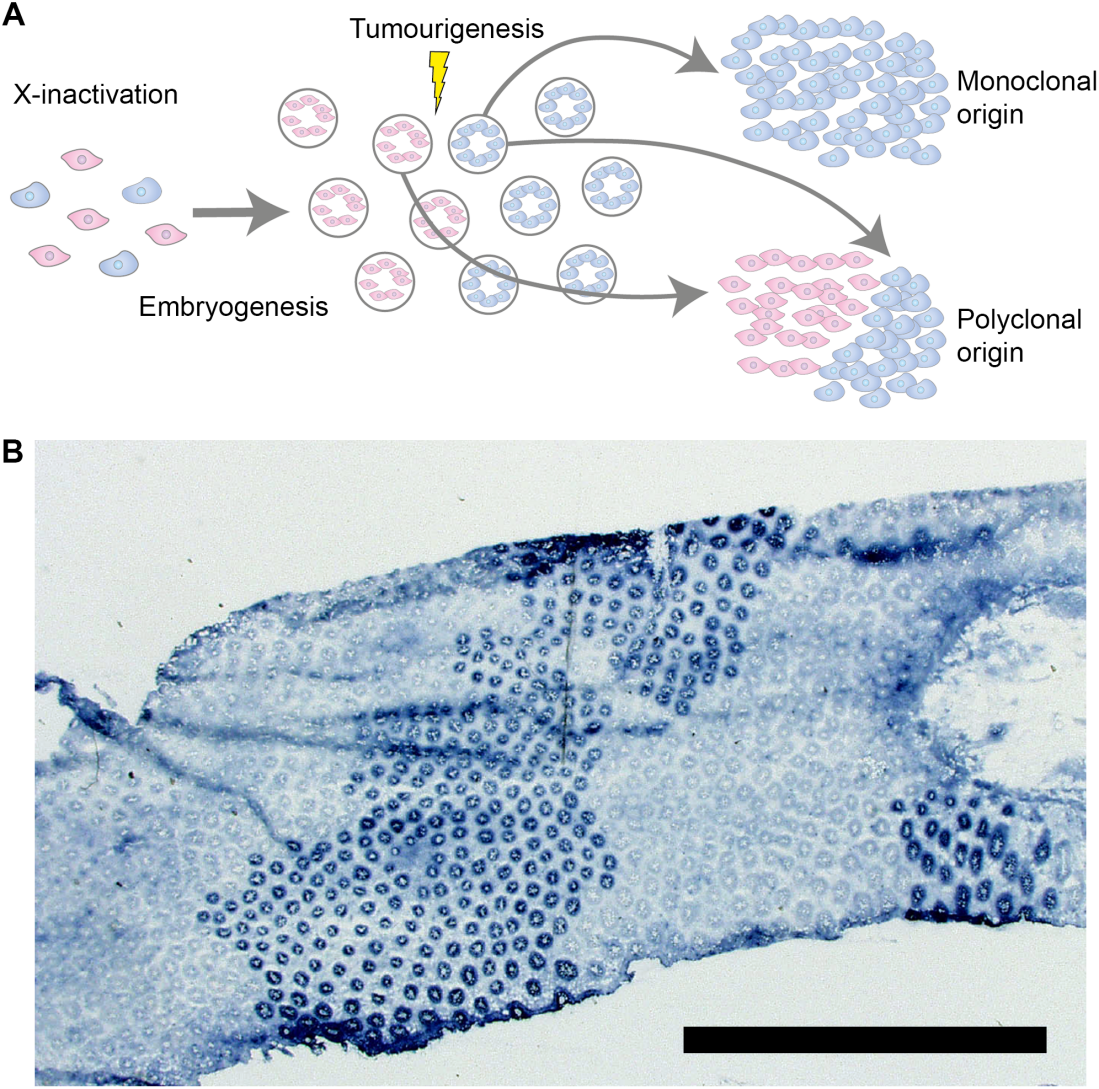
Lineage tracing using germline mutations. (A) An illustration of how germline mutations ‘label’ a cell lineage. As an example of this, early in development, rare individuals with sex‐linked germline mutation inactivate either the wildtype or mutant allele, labelling the diverging lineages. Following embryogenesis, the mutant and wildtype lineages are evidenced as large, contiguous clonal patches. The relative mixing (or lack thereof) of these two genotypes is therefore informative about the clonal makeup of the constituent tissue. An underappreciated facet of these sorts of studies is that they are only powered to detect mixing at the boundaries of clonal patches. (B) A top‐down slice of colon epithelium stained for G6PD activity. The mutant crypts form large clonal patches, reflecting the X‐inactivation of a particular cell lineage early in development. Reproduced with permission from [18]. Copyright (2003) National Academy of Sciences, U.S.A.

The very first lineage tracing techniques applied in human exploited X‐inactivation (also termed Lyonization) of sex‐linked genes. In females, one copy of the X‐chromosome inactivates during embryogenesis by DNA methylation that silences gene expression, meaning that there is not a gene dosage asymmetry between males and females. The selection of which X‐chromosome is silenced is random; hence, the cells of females with a heterozygous polymorphism located on the X‐chromosome will have an approximately equal probability of expressing either phenotype (Figure 1A). In 1965, Linder and Gartler [19] exploited germline heterozygous mutations in the glucose‐6‐phosphate dehydrogenase (G6PD) gene that abrogate expression to show that, whilst their normal uterine cells were a hodgepodge of cells expressing one of the two alleles (notionally the A and B alleles), all the cells in tumours from these patients expressed either the A or B alleles (but not both). At the time, this was seen as compelling evidence that cancer arose from a single cell, rather than a collection of cells.

More recently, direct visualisation of the different G6PD phenotypes allowed researchers to observe that cells that share the same X‐activation status are found together in large clonal patches because X‐inactivation occurs relatively early in the developmental process, and therefore studies of this type are biased towards finding a monoclonal origin of cancers (Figure 1B) [18]. This highlights a broader point: the timing of when a marker appears during development, or equivalently the rate at which a marker is introduced for the case of continuous labelling (e.g. for ongoing somatic DNA mutations), determines the temporal and spatial resolution at which cell dynamics can be assessed.

As an example of this principle, staining for G6PD activity in mouse colon [20] incorrectly concluded that each colonic crypt is maintained by a single stem cell (more recent lineage tracing studies revealed that crypts are maintained by a pool of equipotent stem cells, as we shall discuss in more detail below). The authors applied a carcinogen over 21 weeks that induced somatic abrogation of G6PD expression, then stained for G6PD expression 2 weeks after the final carcinogen dose. They did not identify any examples of mixed G6PD expression phenotypes, and thus concluded that crypts are maintained by a single stem cell. However, the authors failed to account for the possibility of clonal expansion. If a pool of equipotent stem cells were replacing each other rapidly and the rate of mutation induction was sufficiently low, one would not expect to see a large number of partially mutant crypts. As above, the power of the study to resolve clonal dynamics was limited by the induction rate of the lineage tracing marker.

In blood, germline mutations in X‐linked genes were utilised to probe the haematopoietic stem cell (HSC) pool and time X‐inactivation via an elegant argument arising from binomial statistics [21]. The key concept was that the proportion of blood cells expressing one of the two alleles is reflective of when in the developmental process Lyonization occurred. If this X‐inactivation occurred following the first division of founder HSC, across patients, we would expect all to contain roughly equal proportions of the two X‐inactivation patterns. If instead, Lyonization occurred following the second division when there were four founder cells, we would expect to observe some patients with roughly a quarter of their blood cells as one X‐inactivation pattern and three quarters as the other. Following this logic, the authors compared the cumulative mass probability of the proportion of blood cells with a given X‐inactivation pattern to the expected binomial distribution with 4, 8, and 16 founder cells, concluding that 8 founder cells (i.e. following the third division) best fit the data. Further, the authors went on to estimate the number of HSCs by examining the intrapatient heterogeneity in phenotype at multiple timepoints, estimating that the blood stem cell pool is maintained by 400 HSCs. We note that multiple adult clones are nested within each embryonic clade, so this places only a lower bound on the number of HSCs.

As with heterozygous sex‐linked germline mutations, individuals with other rare genotypes offered early researchers' natural markers to track lineages within normal and aberrant tissue. A prime example of this is an individual with X0/XY mosaicism who coincidentally also had familial adenomatous polyposis (FAP), a hereditary condition in which a heterozygous germline *APC* mutation leads to a heightened risk of colorectal cancer [22]. *In situ* hybridisation (ISH) allowed for visualisation of the X0/XY phenotype and revealed that morphologically‐normal intestinal crypts were composed of exclusively one of the two phenotypes arranged into irregular clonal patches, strongly suggesting the clonal origin of intestinal crypts. As expected, villi at the border of the two phenotypes were a mixture of X0 and XY, confirming that villi were fed from multiple crypt populations. Intriguingly, several adenomas contained a mixture of X0 and XY cells, suggesting a polyclonal origin of these precancerous lesions; although subsequent statistical analysis cautioned that the authors overestimated the polyclonal fraction [23].

Another naturally occurring lineage tracing marker is O‐acetylation of sialoglycoproteins in goblet cells, which can be distinguished from non‐O‐acetylated sialoglycoproteins with mild periodic acid‐Schiff (mPAS) staining [24, 25]. The OAT gene determining O‐acetylation is autosomal; hence, in individuals heterozygous for OAT, the majority of colonic crypts are mPAS‐negative (O‐acetylated) but with sporadic mPAS‐positive crypts randomly distributed across the colon, the frequency of which increases with age as the result of somatic mutation [26]. The OAT mutation rate is greatly increased during radiotherapy, leading to a dramatic increase in the number of mPAS‐positive crypts following treatment. Notably, immediately following treatment, a large number of crypts that are a mixture of mPAS^+^ and mPAS^−^ cells are observed, but over time the number of partially fixed crypts falls [27]. This allowed Campbell *et al* to estimate that the time to monoclonal conversion of human colonic crypts following radiation is ∼1 year [27], significantly longer than the few months that had been previously estimated for mouse [28, 29]. Importantly, unlike G6PD staining, mPAS staining can be performed upon formalin‐fixed paraffin‐embedded (FFPE) tissue.

## Mitochondrial DNA mutations

While heterozygous germline mutations provide powerful tools to explore clonal relationships between cells, it limits our field of view to rare individuals who carry these alterations, and is only powered to detect clonal differences at the boundaries of large clonal patches. Leveraging the multiple somatic mutations that occur during ageing, rather than the few germline/early developmental mutations, would allow for more recent clonal architecture to be resolved. Until the recent advent of sensitive whole‐genome sequencing methods [30], it was generally infeasible to use somatic nuclear DNA mutations as markers. This was because genomic analysis was restricted by the use of targeted (Sanger) sequencing, which could analyse only a few hundred base pairs of DNA in a single sequencing run; given the low somatic mutation rate, the probability of a somatic mutation would exist within these small genomic regions was incredibly low. To circumvent this technical hurdle, the second generation of lineage‐tracing techniques instead focused on alterations that occurred at a higher rate than nuclear DNA mutations, namely, mutations of mitochondrial DNA (mtDNA).

Naturally occurring, somatic mtDNA mutations accrue at a mutation rate orders of magnitude greater than nuclear DNA mutations, and are therefore more likely to be present in a sample (reviewed in [31]). Nevertheless, mtDNA mutations occur infrequently enough that the odds of the same mutation occurring independently in two cells in close spatial proximity is low. MtDNA mutations can therefore be used directly to trace somatic cell lineages.

**Figure 2:**
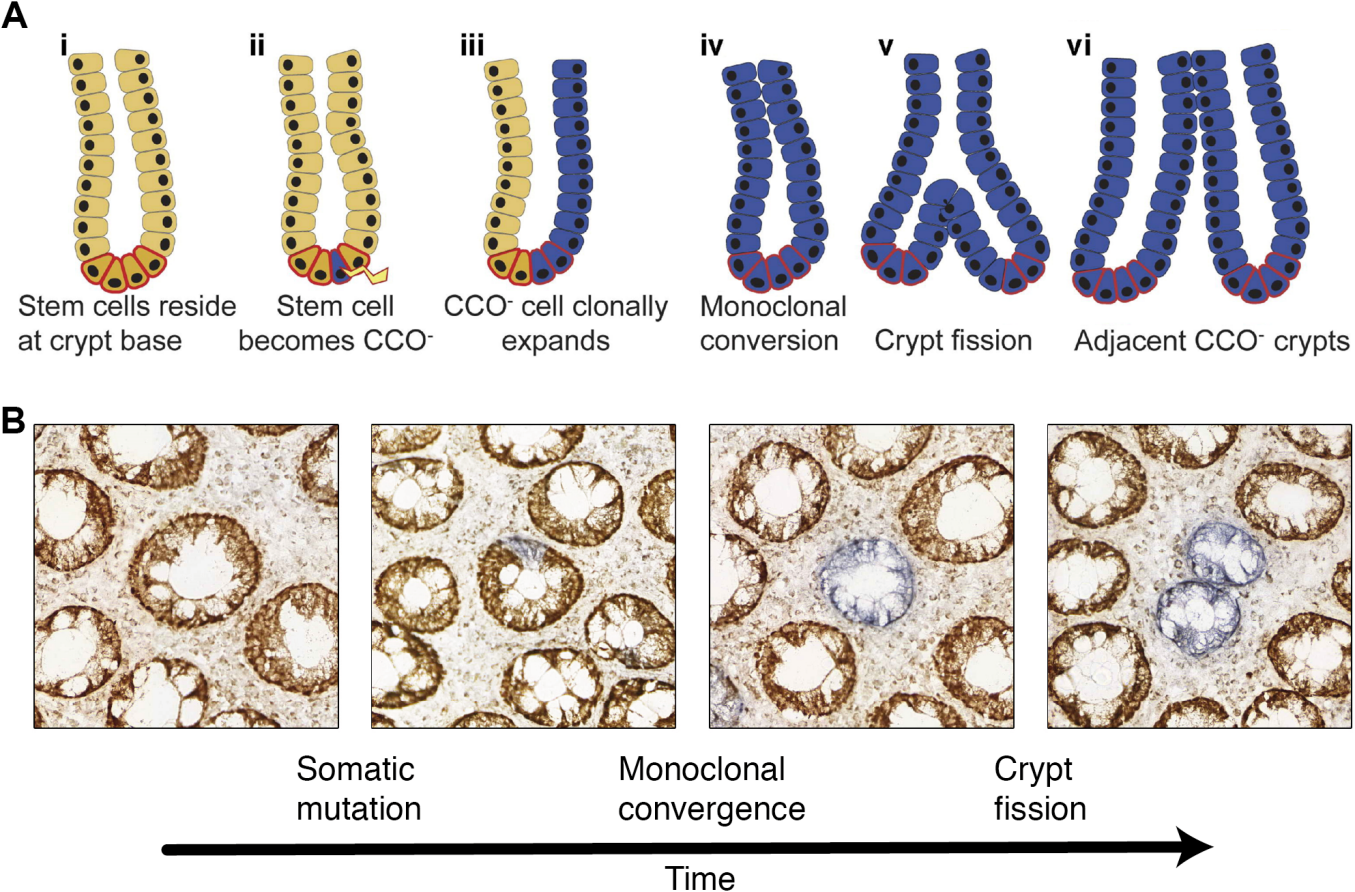
Ongoing somatic mutation as lineage markers. (A) An illustration of how ongoing somatic mutations enable lineage tracing of dynamic systems. At birth, all cells in a given tissue are wildtype to a specific somatic mutation. Over time, these somatic mutations can occur in individual stem cells. If the mutant stem cell undergoes clonal expansion, all the progeny of that cell will also be labelled. Due to the low rate of somatic mutation, the probability that two stem cells in close proximity will have independently developed the same mutation is low. Because somatic mutations are ongoing, they have the opportunity to resolve more recent clonal expansions. Reprinted with permission from [32]. Copyright (2011), AGA Institute. (B) Representative examples of CCO deficiency of crypts in the colonic epithelium which allow clonal expansions to be visualised. A stem cell within a crypt that is initially wholly wildtype (brown) first undergoes an mtDNA mutation (blue). By a process of neutral drift, this labelled stem cell can expand and fix within the niche. A somatic mutation that has fixed within a colonic crypt can then spread by crypt fission, forming a clonal patch.

Importantly, in addition to the raw genetic information in mtDNA mutations can also lead to histochemically‐detectable changes in protein expression, allowing direct visualisation of clonal relationships *in situ* in human tissues. There are multiple copies of a given mtDNA gene in a single cell, but once a mutation has occurred in a single mitochondrial genome, there is a chance of that mutation coming to dominate the cell via genetic drift, leading to that cell expressing the mutant phenotype. A pertinent example is the loss of expression of the mitochondrially‐encoded gene cytochrome *C* oxidase (CCO), readily visualisable via histochemical staining (Figure 2), which is caused by an underlying mtDNA mutation that has expanded to become dominant in the mtDNA pool.

Such mtDNA markers were used with great success to map the clonal dynamics of intestinal stem cells. Studying colon tissue from older individuals, Taylor and colleagues observed that crypts were either CCO‐proficient (CCO+), wholly CCO‐deficient (CCO−), or a mixture of the two phenotypes [33]. These experiments demonstrated that somatic mutations first occur in a single stem cell within a crypt (causing a partial CCO− crypt), before clonally expanding until the mutant allele reaches fixation in a process termed monoclonal conversion (causing a wholly CCO− crypt); hence, confirming that intestinal crypts are maintained by a pool of stem cells, rather than a single asymmetrically dividing stem cell. Our subsequent work recognised that the number of wholly CCO− mutant crypts increases with age and that CCO− crypts cluster in small patches where adjacent CCO− crypts all carry the same mtDNA mutation [34]. Together with an analysis of bifurcating crypts, where the same CCO− mutation was present in both crypt arms, these lineage‐tracing data provided clear evidence of the clonal expansion of human colon crypts by the process termed crypt fission (reviewed in [32], Figure 2A) [35, 36].

Somatic mtDNA mutations have been applied to uncover the clonal architecture of the liver [37], stomach [38], breast [39], oesophagus [40], prostate [41, 42], and bladder [43].

More recently, the advent of high‐throughput single‐cell sequencing has extended the possibilities for mtDNA‐based lineage tracing. In chromatin accessibility sequencing (assay for transposase assessable chromatin: ATACseq), a transposase is applied to the DNA of interest, which cuts and inserts a sequencing adapter in regions of open (accessible) chromatin. MtDNA lacks chromatin structure and so is accessible and thus labelled by the transposase. In single‐cell ATACseq (scATACseq), upon sequencing, thousands of reads are derived from the multiple mtDNA copies present in each cell providing sensitive detection of the somatic mtDNA mutations present in each cell [44, 45]. These high‐throughput techniques offer utility in addition to that provided by our previous histology‐based approach because they allow very large numbers of cells to be ‘screened’ for mtDNA mutations, enabling the detection and characterisation of clonal relationships even in the absence of histologically evident clonal expansion.

## Mathematical analysis of single‐label lineage‐tracing data

**Figure 3:**
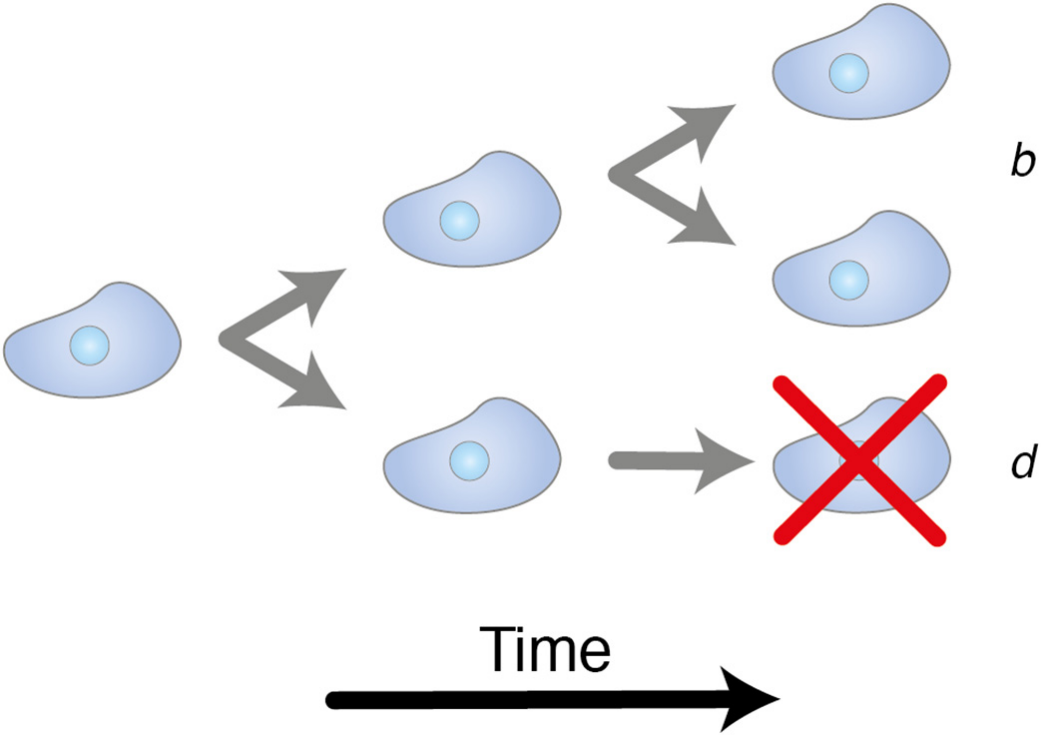
Branching processes. An illustration of the basic structure of branching models. Over time, each cell within a population stochastically either divides at a rate b, increasing the population, or dies at a rate d.

A commonly used mathematical formulation to describe the dynamic process of cell birth and death are branching models. In these models, a cell can either divide to form two daughter cells at a rate b, growing the population, or be removed from the population (die) at a rate d. At each birth step, the phylogenetic tree that describes the evolutionary relationships between cells branches (Figure 3), hence the name. Branching models provide a flexible framework for modelling evolution, allowing for the potential inclusion of mutations and multiple cell ‘types’ (e.g. labelled and unlabelled members of the population, or cells with a selective advantage). An excellent introduction to branching processes can be found in Jones and Smith [46].

Using statistical inference techniques, we can discover the parameters of the mathematical model that make the model reproduce the key features of observed (biological) data. For an example, we can construct a model that describes the emergence and expansion of CCO− clones in the colon, and fit this model to data we have collected on the distribution of CCO− patch sizes we have observed across colonic resection specimens [47], resulting in the inference of the rate of crypt fission. A very important consequence of interpreting data with dynamical mathematical models is that we can derive quantitative information about dynamic processes, even though our data are collected at only a single point in time.

Mathematical models describing colon stem cell evolution provide an instructive example to delve deeper into the mathematics of lineage tracing. A mathematical model for continuous labelling at rate ω of S stem cells arranged in a ring replacing each other at a rate λ per stem cell per division was explicated by Kozar *et al* [48]. In the long‐time limit, the model predicted that the fraction of fixed mutant crypts will increase approximately linearly with time, t ($C_{\text{fixed}}=\mathrm{\omega t}$, although this expression fails to account for the time taken for a mutant stem cell to fix within the crypt); whereas the fraction of partially fixed crypts tends towards a constant value ($C_{\text{partial}}=\frac{\mathrm{\omega S}\left(S-1\right)}{2\lambda }$). Thus, the ratio of the two fractions is independent of the mutation rate, $\frac{C_{\text{fixed}}}{C_{\text{partial}}}=\frac{2\lambda }{S\left(S-1\right)}t$. The intuition behind the expression for $C_{\text{fixed}}$ can be understood by considering that the mutation rate per cell is ω. The rate of any one of the S stem cells developing a label is thus ωS, but each stem cell only has a $\frac{1}{S}$ chance of fixing within the crypt; hence, the factor of S cancels. Conversely, the number of newly induced partially labelled crypts is compensated for by the ongoing clonal extinction/fixation events, and thus $C_{\text{partial}}$ is a constant depending on the replacement rate and the number of stem cells. The ratio of the two fractions depends both on the stem cell number and the replacement rate, hence this ratio alone is insufficient to separately identify λ and S. Additional data that measures the rate of lineage labelling (which ultimately sets the number of fixed crypts that are expected to be observed) is needed to separately identify the clonal dynamics.

Utilising this theory, Stamp *et al* [49] and Nicholson *et al* [50], applied histoenzymatic staining to visualise somatic mtDNA and DNA mutations (specifically, OXPHOS‐deficiency and loss of O‐acetylation of sialomucins, respectively). By counting the number of partially and fully fixed mutant crypts from individuals with a range of different ages, they estimated the cohort average replacement rate and stem cell number. Together, these studies implied that the vast majority of stem cell divisions in human are effectively asymmetric, producing one cell that remains as a stem cell whereas the other differentiates. Rare symmetric divisions, producing two cells that remain as stem cells and displace another stem cell lineage from the niche, lead to long average times to fixation.

Mathematical models have also been instructive in measuring the rates of crypt fission in the human colon, and the recently recognised counteracting process of crypt fusion (whereby two adjacent crypts fuse to form a single descendant [51]). As noted above, CCO− crypts are found in increasingly large patches in older individuals [34], and the size of these patches is evidently dependent on the rates of crypt fission and fusion. Fitting the observed distribution of CCO− patch sizes to an appropriate mathematical model [52] reveals that crypt fusion occurs at approximately the same rate as crypt fission, roughly once every 90 years per crypt, suggestive of its role in homeostasis. This finding resolved a longstanding biological puzzle, in which it was known that crypt fission provided a mechanism for new crypts to enter the population, but that the total length of the colon and the density of crypts appear to remain largely constant over time.

## Somatic epimutations

**Figure 4:**
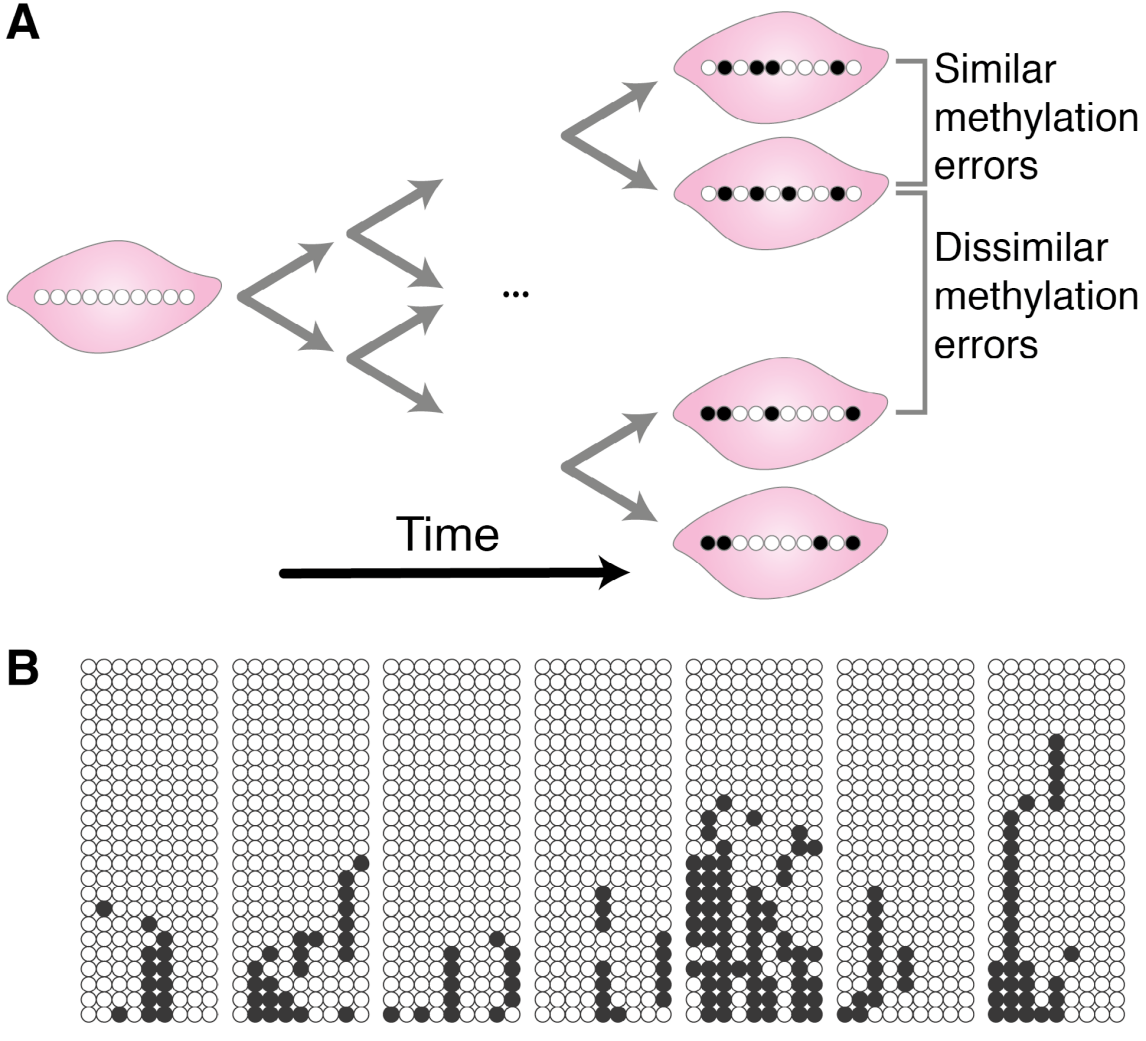
DNA methylation as genetic barcodes. (A) An illustration of how somatic changes in methylation can be employed as genomic barcodes. At birth, certain CpG loci are unmethylated in every cell (white circle), but over time these CpG loci can spontaneously become methylated (black circle) in individual cells. The progeny of these cells carry the same pattern of methylation changes. Hence, the epigenetic distance between cells serves as a proxy for their relatedness. (B) Example methylation barcode data. Each circle represents a single methylated (black) or unmethylated (white) CpG locus, each row of circles corresponds to a single methylation tag, and each block represents a set of methylation tags from a single crypt, all taken from the colon of a 58‐year‐old individual. The methylation patterns within a single crypt are more similar to each other than the methylation patterns between crypts, due to the recent niche succession within the crypt. Reproduced with permission from [54].

An alternative somatic lineage‐tracing technique that we developed was based on epigenetic changes to the DNA, principally changes in DNA methylation (hereafter referred to as ‘epimutations’) [53]. DNA methylation changes much more rapidly than the DNA sequence itself, whilst still being somatically heritable. If one considers the methylation on a single DNA strand, a particular CpG locus can either be methylated or unmethylated. In this way, a set of allele‐specific methylation patterns from a single clone can be considered as a binary string (referred to as a ‘tag’ or ‘barcode’) of 1's (methylated) and 0's (unmethylated). CpG loci located on CpG islands are typically unmethylated at birth and the methylation level of a number of these CpG loci increase approximately linearly over time (Figure 4A). CpG loci that follow this pattern can be selected by searching for those loci where the percentage of methylated sites increases with age. Clonal relationships within and between clonal patches can be assessed both via the diversity in unique barcode tags found within and between individual clonal patches and by the Hamming distances (the proportion of CpG sites where the methylation status differs between two molecules) between the unique barcodes (e.g. Figure 4B). A number of studies have found that DNA methylation reflects clonal ancestry, in both normal development [55] and cancer [56, 57, 58].

In the context of intestinal crypts, methylation barcodes were first employed to test the then competing hypotheses of immortal stem cell versus the stem cell niche. In the immortal stem cell hypothesis, a crypt was proposed to be maintained by multiple stem cell lineages that all divided strictly asymmetrically, with the differentiated daughters producing the rest of the crypt cell population [59]. Contrastingly, in the niche model, a crypt is maintained by a population of multipotent stem cells that compete with each other to retain their place in the niche, and so the stem cell lineages undergo stochastic loss and replacement. One would expect that if intestinal stem cells were immortal, there would be a greater degree of variability in the intracrypt methylation pattern diversity than that predicted by the niche hypothesis. This is because, in the niche model, one stem cell will inevitably clonally expand until it has replaced all other stem cells in the niche, generating a new common ancestor. Our single‐molecule resolution sequencing of select CpG loci, again interpreted with mathematical modelling, provided evidence that intestinal crypts in human were maintained by multiple stem cells competing for their place in the niche, rather than a number of immortal stem cells [5, 53]. Further, we observed that methylation barcodes were similar between the bottom and top of each crypt, confirming that the mutations present in the differentiated cells reflect that of the stem cell ancestor.

We subsequently applied methylation lineage tracing to other tissues, including hair [60], endometrium [61], and different immune cell lineages in the blood [62]. Intriguingly, unlike in the colon, the average methylation error levels of hair follicles do not increase with age, suggesting that the bulge stem cells divide only infrequently, with the bulk of the methylation errors accumulated in long‐lived but mortal transit‐amplifying cells. Conversely, average methylation in the endometrium does increase with age until ∼50 years, at which point it plateaus due to the decrease in the cell division rate following menopause. In blood, different leucocytes experienced varying rates of epigenetic error accumulation according to each cell type's position on the differentiation hierarchy. Together, these studies demonstrate the broad‐ranging applicability of methylation barcodes as molecular clocks.

More recently, methylation barcoding techniques were used to compare chronic lymphocytic leukaemia (CLL) to healthy B‐cells [63]. The authors found that, despite the CLL cells displaying a greater epimutation rate, indicating increased tissue aging, the cell‐to‐cell variability in epimutation rates was lower than in healthy B‐cells, reflecting the common ancestry of the cancer. Phylogenies built upon the methylation data revealed that CLL had balanced trees, consistent with CLL evolving under a neutral‐drift paradigm following malignant expansion.

Whilst methylation barcoding presents a powerful technique to resolve clonal dynamics, it is not without its technical limitations. Bisulphite conversion, which converts unmethylated cytosine to uracil and is the standard method to determine the methylations patterns across the genome, is a destructive process causing DNA degradation, and careful optimization is required for low DNA input amounts. Fortunately, bisulphite‐free methods for characterising methylation have been proposed [64, 65], although these methods are not yet standard practice.

## Clone‐by‐clone analysis

A subtle but important limitation of the lineage‐tracing data described thus far is that inferences are made from the complete ensemble of data, giving average behaviours rather than clone‐specific measurements. In other words, from CCO− patch size data, for instance, we can infer the average rate of crypt fission, but not the fission rate of an individual patch.

**Figure 5:**
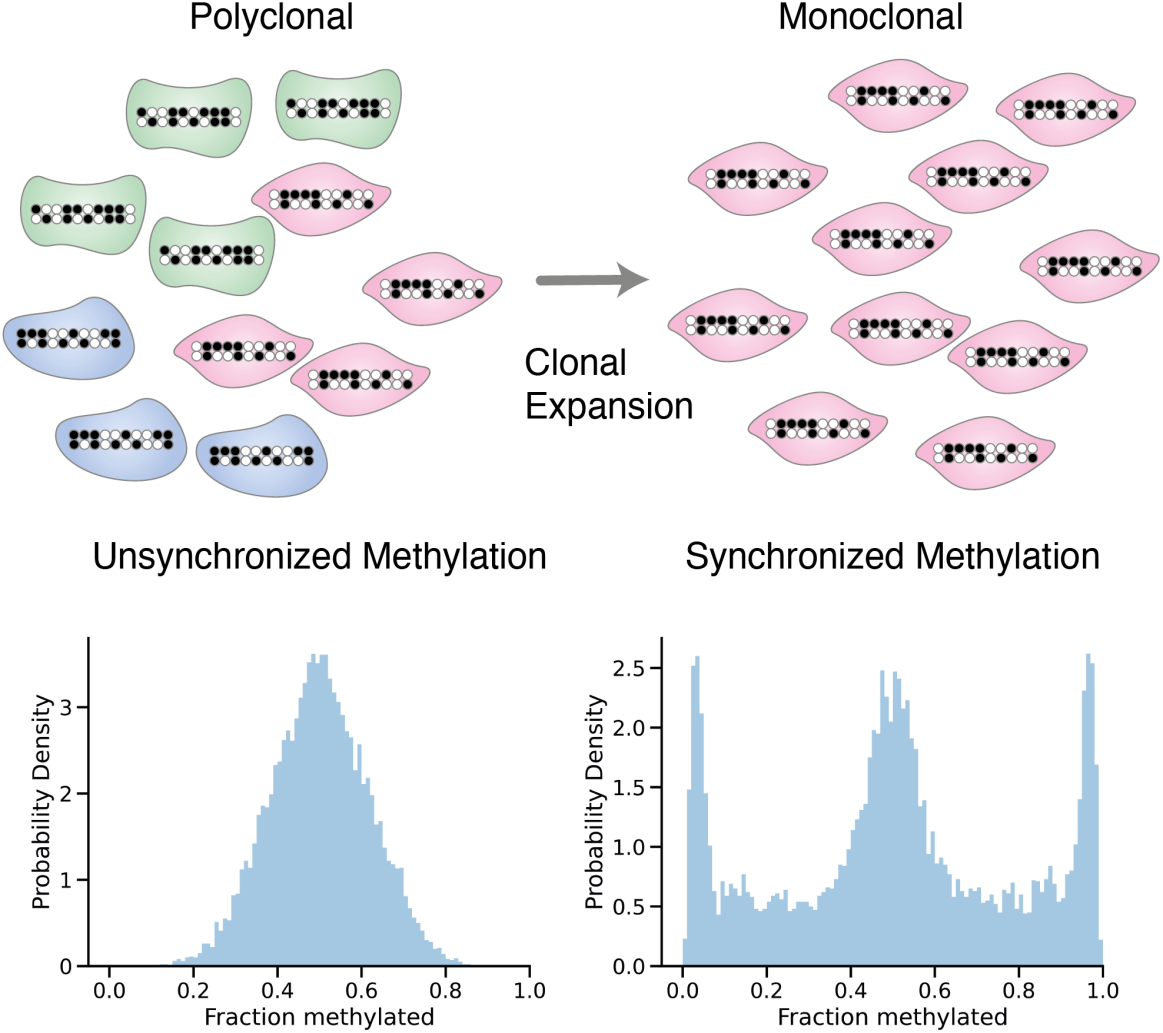
Clonal expansion synchronises FMCs. In a large polyclonal population, the unsynchronised fluctuating methylation patterns average out to ~50% methylated, leading to a histogram of the methylation level yielding a unimodal distribution (left). However, if a population undergoes a clonal expansion, the methylation patterns of the resulting population inherit that of the progenitor cell, effectively synchronising the methylation clocks and yielding a distinctive W‐shaped distribution (right).

We recently developed a novel lineage‐tracing method based on fluctuating methylation clocks (FMCs), which enable clone‐by‐clone individual measurements of stem cell dynamics [66]. The key difference compared to previous methods is that selecting for fluctuating CpG (fCpG) loci that stochastically jump between homozygously methylated, heterozygously methylated and homozygous demethylated states in individual diploid cells allows for recurrent clonal dynamics to be measured. In stem cell pools with rapid clonal expansion and fixation, the distribution of bulk methylation patterns will reflect that of the recent progenitor cell, leading to the FMC distribution of individual colon crypts bearing a characteristic ‘W‐shape’ (Figure 5). Contrastingly, the methylation states of fCpG loci in polyclonal populations will be desynchronised, such that the FMC distribution is unimodal. In this manner, the FMC distribution encodes the clonal dynamics of a stem cell population. We developed a mathematical model to link the number of stem cells, their replacement rates, and the rates of fCpG (de)methylation to the measured FMC distribution, allowing us to infer these clonal dynamics with readily‐available microarrays.

Unlike previous attempts to measure intracrypt dynamics in humans [49, 50], which relied on measurements across a cohort with a wide range of ages, the FMC method works on individual clonal structures, allowing the intraindividual heterogeneity of clonal dynamics to be probed. This allowed us to conclude that the intraindividual variability in the effective number of stem cells was significantly greater in the endometrium than the colon, perhaps reflective of the dynamic nature of the endometrium through menstrual cycles and age‐related changes. Furthermore, in the same study we demonstrated that the fluctuating methylation lineage tracing approach applies across a range of tissue types and is not only useful for measuring the random neutral dynamics underlying homeostasis, but also for probing the rapid growth of malignant populations. This method, and others like it, which combine novel mathematics with lineage tracing markers, are powerful tools for measuring dynamic somatic cell evolution from a single snapshot in time.

## Whole‐genome sequencing as a lineage‐tracing tool

The advent of next‐generation sequencing (NGS) has allowed for deep whole‐genome sequencing (WGS) of multiple tissue samples from the same individual to be performed relatively cheaply. This enables the use of somatic nuclear DNA mutations as lineage‐tracing markers, because approximately 1–10 DNA mutations occur in every cell division [12, 13, 14], meaning every cell is uniquely marked. Of course, even with high depth sequencing, mutations that have occurred very recently will only be present in a small fraction of cells (unless that mutation is strongly selected for and has therefore undergone a clonal sweep). In this way, the link between mutation rate, depth of sequencing, and the temporal resolution of lineage tracing markers is evident; higher depth sequencing allows one to resolve more recent clonal dynamics, whilst faster mutation rates are more likely to ‘catch’ a given clonal event and provide more power to distinguish between ancestries. There is also an interrelationship between the size of a tissue sample (number of cells) and the clonality of that sample. Subclonal mutations will likely be at higher frequency in samples with fewer cells (as there are fewer ‘non‐clonal’ cells). Samples consisting of cells with a recent common ancestor (e.g. a colon crypt) will have more high‐frequency mutations than a random sample of cells (e.g. from an endoscopic brush). These sampling considerations influence intra‐ and intersample clonality assessment in subtle ways (we refer the reader to the supplementary material of [67] for a mathematical assessment) and care should be taken to choose and/or normalise for the sampling scheme in lineage‐tracing analyses. Further, detecting low‐frequency mutations is a pressing technical problem, in part due to the difficulty of distinguishing between low‐frequency mutations and errors introduced during polymerase chain reaction (PCR) amplification. However, the detection limit of NGS can be improved dramatically with innovative techniques, such as duplex sequencing [68] and applying careful bioinformatic tools to technical replicates [69].

Multiregion WGS has revealed that the mutational landscape of normal tissue is significantly more altered than previously thought [2, 3, 70], with large clonal patches containing key cancer driver mutations evident across tissue types. In the normal colon of middle‐aged individuals, 1% of crypts were found to harbour a known colorectal cancer driver mutation [71]. Work in skin has shown that driver mutations in normal tissue vary with body site [72], suggesting that the spectra of induced mutations and the fitness landscapes of different normal tissues are highly diverse. The power of employing DNA mutations as lineage‐tracing markers is that they allow for inference of the selective advantage of repeatedly observed mutations to be inferred. This has been done principally by considering the mutation rate normalised ratio of nonsynonymous to synonymous mutations (dN/dS). Synonymous mutations are assumed to be neutral, whereas nonsynonymous mutations can experience selection, thus an excess of nonsynonymous mutations is indicative of positive selection (adaption) [73]. Analyses of clone size distributions (or analogous variant allele frequency [VAF] distributions) provide alternative measures of selection [74, 75, 76]. Whilst the initial analysis of the clone size distribution applied to human skin did not find evidence of widespread selection [74], contradicting the conclusions of the original dN/dS approach [2], subsequent analysis reconciled the two [77], indicating that the effect of selection on the clone size distribution can be obscured by spatial constraints and experimental limitations.

Applications of these complementary methods show that some cancer driver mutations, for example, *TP53* and *NOTCH* mutations in the oesophagus [70, 78], arise in healthy tissues as a consequence of both natural ageing and environmental factors [79], and expand due to strong selective pressure [78, 80]. In fact, bizarrely, normal oesophagus appears to bear a higher rate of *NOTCH1* mutation than oesophageal cancer, which led Colom *et al* to suggest a model in which early tumours are outcompeted by *NOTCH* mutant but morphologically normal epithelial cells [81].

WGS has also provided important new insight into HSCs, with recent work [1] applying phylogenetic techniques to multiple WGS sequenced clonally expanded HSCs from a single individual, allowing the authors to infer that blood is maintained by 50,000–200,000 HSCs, which share only a very distant common ancestor. In a contrasting approach, Watson *et al* analysed the VAF distributions of healthy blood samples from ∼50,000 individuals, determining that clonal haematopoiesis is driven by positive selection rather than genetic drift [76].

Widespread WGS of cancerous tissue, together with mathematical models of clonal dynamics, has led to intriguing insights into the evolutionary histories of growing tumours. For instance, a simple mathematical model of a population growing exponentially under effectively neutral dynamics suggests that the cumulative frequency ($M\left(f\right)$) spectrum follows a characteristic $M\left(f\right)∼\frac{1}{f}$ distribution [82]. Turning to data, we observed a good fit of this model in over a third of cancers investigated. Considering tumour WGS data as a form of lineage tracing data and interpreting it with (simple) mathematical models that depict the basic biological processes of cell growth and mutation, allowed us to measure the mutation rates, timing of mutations, and selective advantages of key driver mutations from bulk sequencing data [83]. A particular use of the clone size analysis approach is that it can measure evolutionary dynamics on a patient‐by‐patient basis, whereas the alternative dN/dS approach can only measure cohort level averages (as data must be combined across patients to yield sufficient mutations for the analysis).

Employing genomic mutations as naturally occurring markers has great potential, not only for retrospectively inferring a cancer's history, but also for predicting future evolution. Rather than tracking individual SNVs, large‐scale copy number aberrations (CNAs) also define lineages. CNAs typically occur less frequently than SNVs, but their presence can be detected with much lower depth sequencing, making sequencing significantly cheaper and opening the door for single‐cell analysis. Time series single‐cell WGS (scWGS) allowed Salehi *et al* [84] to infer how the specific burden of CNAs borne by a cell affected determine the clone's relative fitness within the tumour. Once clone‐specific fitnesses had been delineated, the authors could accurately predict which clones would come to dominate the tumour during treatment. In this manner, the genotype can function as a proxy for individual cancer cells' phenotype.

## Concluding remarks

Lineage‐tracing data in human is usually ‘static’, in that it is collected at a single point in time. Mathematical models can be constructed that present the dynamical processes that lead to the clonal structure captured in the data, and fitting the mathematical models to data, to infer the parameters that control model behaviours, enables indirect measurement of the underlying dynamics. Thus, together static lineage‐tracing data interpreted through the lens of mathematical modelling provides a powerful lens into the dynamics of somatic evolution.

Importantly, mathematical models provide both a rational framework to guide experimental design and allow rigorous testing of competing hypotheses against the data (e.g. the immortal versus niche model of colon stem cell dynamics described above). It is worth mentioning that, whilst any particular mathematical model relies on several assumptions, failing to cast a particular result in mathematical terms does not eschew the implicit assumptions that any purely qualitative model rests upon; it simply obfuscates their implications. Arguably, the process of mathematical modelling forces the scientist to be explicit about the assumptions they use to interpret the data and to rigorously consider their consequences for the conclusions drawn.

One of the subtle aspects of employing naturally occurring lineage‐tracing markers is that the mutation (labelling) rate of a particular marker defines the temporal resolution of the biological phenomena that can be studied. That is, if mutations occur only very rarely (as in the case of nuclear DNA), then distinguishing between recent clonal divergence will be difficult, as few mutations will have occurred since the time of divergence. Conversely, if a mutation occurs too rapidly, then over the course of an individual's lifetime the marker will saturate, with unrelated lineages independently developing the same mutation. In the particular case of nuclear DNA, the low mutation rate can be offset to a degree by analysing every base pair (e.g. via WGS), allowing the breadth of the genome to compensate for the slow mutation rate. However, this is limited by both the cost and technical limitations of performing a very high depth of sequencing on small numbers of cells. ‘Relabelling’ techniques, such as our FMC method [66], somewhat side‐step the problem of saturation but sites with methylation that fluctuates at rates comparable to the timescale of the biological process of interest must still be found.

The combination of using somatic (epi)mutations and mathematical modelling provides a powerful toolkit to measure the clonal architecture of tissue structures in human and infer the otherwise unobservable temporal dynamics of cell birth, death, and replacement.

## Author contributions statement

CG wrote the first draft of the review. NW, AB, DB and TAG edited the review. All authors approved the final draft of the article.
